# Generation of a Murine Model for c-MYC and BCL2 Co-expression B Cell Lymphomas

**DOI:** 10.3389/fonc.2020.01007

**Published:** 2020-06-30

**Authors:** Zhenming Cai, Le Zhang, Min Cao, Yuliang Wang, Feng Wang, Weiqi Bian, Sulan Zhai, Xiaoming Wang

**Affiliations:** ^1^Department of Immunology, Key Laboratory of Immune Microenvironment and Diseases, Nanjing Medical University, Nanjing, China; ^2^Analysis Center, Nanjing Medical University, Nanjing, China; ^3^State Key Laboratory of Reproductive Medicine, Nanjing Medical University, Nanjing, China

**Keywords:** murine model, c-MYC, BCL2, Co-expression, B cell lymphomas

## Abstract

Diffuse large B-cell lymphoma (DLBCL) is the most frequent lymphoma in adults, and is characterized as clinically and biologically heterogeneous lymphomas with diverse response to therapy and variation in clinical behavior. It's well-established that c-MYC and BCL2 play important roles in normal B-cell differentiation and tumorigenesis. B cell lymphoma with dual expression of c-MYC and BCL2 (double-expressor lymphoma, DEL) accounts for approximately one-third of DLBCL cases. DEL patients have poor outcomes after chemoimmunotherapy or autologous stem-cell transplantation. Lack of a genetic mouse tool for DEL hinders us from understanding the lymphogenesis mechanism and developing therapeutic strategies. Here, we investigated whether ectopic expression of c-MYC and BCL2 in different stages of B cells could lead to lymphoma and generate a mouse model for DEL. We observed that Co-expression of c-MYC and BCL2 in germinal center (GC) B cells, or pan-B cells could induce B cell lymphomas. The tumor-bearing mice have enlarged lymphoid organs, and B cells massively infiltrate into non-lymphoid organs including lung, liver and kidney. The tumor-bearing mice also manifested significantly shorter lifespan than the controls. In addition, adoptive transfer of Co-expression B cells leads to B cell lymphoma and host mice death. This model will provide us a tool to further explore the pathogenesis and treatment approaches for DEL.

## Introduction

Diffuse large B-cell lymphoma (DLBCL) is the most common type of non-Hodgkin lymphoma (NHL) in the USA, and it accounts for 24% of newly diagnosed NHL cases each year (1). Chemoimmunotherapy with rituximab, cyclophosphamide, doxorubicin, vincristine, and prednisone (R-CHOP), which leads to cure more than half patients, is the most common up-front treatment for DLBCL. However, for the patients who are refractory to up-front treatment, or relapse after achieving remission, the outcomes are particularly poor (2).

It's well-proven that c-MYC and BCL2 play important roles in normal B-cell differentiation and tumorigenesis by affecting different cellular processes (apoptosis, proliferation, cell-cycle control, growth, cell migration, and metabolism) (3, 4). DLBCLs are phenotypically and genetically heterogeneous. Gene-expression profiling has identified subgroups of DLBCLs (activated B cell like [ABC], germinal center B cell like [GCB], and unclassified) according to cell of origin (5, 6). In the revised 2016 World Health Organization classification of lymphoid neoplasms, Co-expression of c-MYC and BCL2 was considered as a new subgroup and defined as double-expressor lymphoma (DEL), which accounts for approximately one-third of DLBCL cases (5). DEL patients with aggressive B cell lymphomas have poor outcomes after standard up-front treatment (7). And DEL is also associated with inferior outcomes after autologous stem-cell transplantation in patients with relapsed or refractory DLBCL (8). Therefore, DEL mouse model is urgently needed to study in-depth mechanism of pathogenesis and develop new therapeutic approaches.

Here, we investigate whether Co-expression of c-MYC and BCL2 in B cells could generate DEL genetic murine models. We show that Co-expression of c-MYC and BCL2 in germinal center (GC) B cells or pan-B cells could induce B cell lymphoma. The tumor-bearing mice have enlarged spleen, mesenteric lymph node (mLN), and peripheral lymph node (pLN), and B cell expansion in the lung, liver and kidney. And the tumor-bearing mice show significantly shorter lifespan than the controls. In addition, adoptive transfer of Co-expression B cells leads to B cell lymphoma and host mice death. The establishment of this model may provide a valuable tool to study the pathogenesis and treatment of *c-MYC* and *BCL2* double-expressor lymphoma.

## Materials and Methods

### Generation of Conditional c-MYC and BCL2 Knockin Mice

All mice were housed in a specific pathogen-free environment in the Animal Core Facility of Nanjing Medical University. The animal protocols were reviewed and approved by the Institutional Animal Care and Use Committee of Nanjing Medical University.

The *c-MYC* (GenBank accession number: NM_010849.4) and *BCL2* (GenBank accession number: NM_009741.5) knockin floxed mice were generated with CRISPR/Cas-mediated genome engineering by Cyagen Biosciences (Guangzhou) Inc. In brief, the “mouse Myc-P2A-Bcl2-polyA” cassette was cloned into intron 1 of ROSA26, and a CAG-LoxP-stop-LoxP was placed upstream of the cassette such that the expression of Myc-P2A-Bcl2 cassette will be dependent on the expression of Cre recombination. To engineer the targeting vector, homology arms were generated by PCR using BAC clone from the C57BL/6J library as template. Cas9 and gRNA were co-injected into fertilized eggs with donor vector for konckin mice production (Supplementary Figure 1A). And the genotypes were identified by PCR (Supplementary Figure 1B). Mice were maintained on a C57BL/6J background. The AID-Cre transgenic mice were kindly provided by Dr. Meinrad Busslinger. B6-CD45.1 (Ptprca Pepcb/BoyJ), B6(C57BL/6J) and CD79a-Cre (Mb1-Cre) mice were purchased from The Jackson Laboratory. Transgenic heterozygote mice (AID^+^ ki/+ refer to GC B cell c-MYC and BCL2 Co-expression mice, and Mb1^+^ ki/+ refer to pan-B cell c-MYC and BCL2 Co-expression mice) were studied and compared with non-transgenic littermates (WT) reared under identical conditions. All mice were sacrificed on 8–10 week, whereas spleen B cells transferred mice were sacrificed on 16 week since the transfer of B cells.

### Flow Cytometry

Lymphocytes were isolated from mouse spleen, mesenteric lymph node (mLN), peripheral lymph node (pLN), thymus and peripheral blood as described previously (9). Liver, lung and kidney were minced, and incubated in 100 μg/ml liberase (Roche) and DNAse I (Roche) at 37°C for 1 h in RPMI 1640 medium with 2% newborn calf serum. A single-cell suspension was prepared by passing the tissue through a 70-μm filter. Lymphocytes from lung, liver and kidney were enriched with 40% Percoll (10).

For GC B cell staining, the following antibodies were from Bio-Legend: anti–B220-APC-Cy7 (RA3-6B2), anti–CD95-PE-Cy7 (Jo2), anti–GL7-FITC (GL7), and anti–CD45.1–PE (A20). Anti–CD45.2-Pacific blue (104) was from eBioscience, and FITC labeled Peanut Agglutinin (PNA, FL-1071) was from Vector.

### Histopathological and Immunohistochemical Examination

In brief, after routine fixation with 4% paraformaldehyde for 24 h and paraffin embedding, tissue sections from mice organs (spleen, lung, liver and lung) were cut (5 μm) and stained with H&E.

For immunohistochemical examination, the slides were deparaffinized with xylene and antigen retrieved in pH 6.4 citrate buffer at 95°C for 20 min, and the endogenous peroxidase activity was blocked by 3% hydrogen peroxide (H_2_O_2_). The following antibodies were used: anti-B220-FITC (Bio-Legend, RA3-6B2), anti-Ki67-FITC (Bio-Legend, 16A8), FITC labeled Peanut Agglutinin (Vector, FL-1071) and CD3 (Vector, VP-RM01). Then the slides were blocked with 3% BSA for 60 min at room temperature and incubated with primary antibody (1:200) at 4°C overnight. Indirect immunohistochemistry was performed with horseradish peroxidase conjugated IgG fraction monoclonal mouse anti-FITC secondary antibody (for B220, Ki67, and PNA, 1:500) (Jackson ImmunoResearch) or horseradish peroxidase conjugated donkey anti-goat IgG (H+L) (for CD3, 1:500) (Jackson ImmunoResearch), and developed by DAB color substrates (Vector Laboratories). Then, the slides were counterstained with hematoxylin. All slides were scanned using a Zeiss Mirax Slide Scanner.

### Spleen B Cells Transfer

Four million spleen B cells (B220^+^) from tumor-bearing or WT mice were injected into 8-week CD45.1 hosts (BoyJ; Jackson Lab). Blood was withdrawn every 2 weeks after B cells transfer and stained with GC B cells as described above. Sixteen weeks after B cells transfer, the host mice were sacrificed and the GC B cells from spleen, mLN, pLN, thymus, lung, liver, and kidney were analyzed by flow cytometry. The spleen, lung, liver and kidney of host mice were also analyzed by histopathological and immunohistochemical examination as described above.

### Western Blot Analysis

B220^+^ B cells were sorted from the spleen of AID^+^ ki/+ and WT mice, or Mb1^+^ ki/+ and WT mice. Then the RIPA extracts were fractionated on 10% sodium dodecyl sulfate (SDS) polyacrylamide gels, electroblotted to polyvinylidene difluoride (PVDF) membranes and reacted with anti-c-MYC (Cell Signaling Technology, 9402), anti-BCL2 (Cell Signaling Technology, 3498) and anti-β-actin (Cell Signaling Technology, 4967) antibodies, and followed by HRP linked goat anti-rabbit antibody (Cell Signaling, 7074). HRP activity was determined using Immobilon Western Chemiluminescent reagent (Millipore, P90720).

### Statistics

GraphPad Prism 8.0 software was used for statistical analyses. The statistically significant differences between groups are assessed by One-way analysis of variance paired with unpaired two-tailed *t*-test. Kaplan-Meier event-free survival curves were generated using the GraphPad Prism 8 software, and statistical significance was calculated using the log-rank (Mantel-Cox) test. Detailed information of the statistical test, number of replicates and number of animals (defined as n) used in each experiment are shown in the figure legends. No randomization was used in the study. *P*-values are denoted in figures by ^*^P < 0.05; ^**^P < 0.01; ^***^P < 0.001.

## Results

### Co-expression of c-MYC and BCL2 in Germinal Center B Cells Induced B Cell Lymphoma

**Figure 1:**
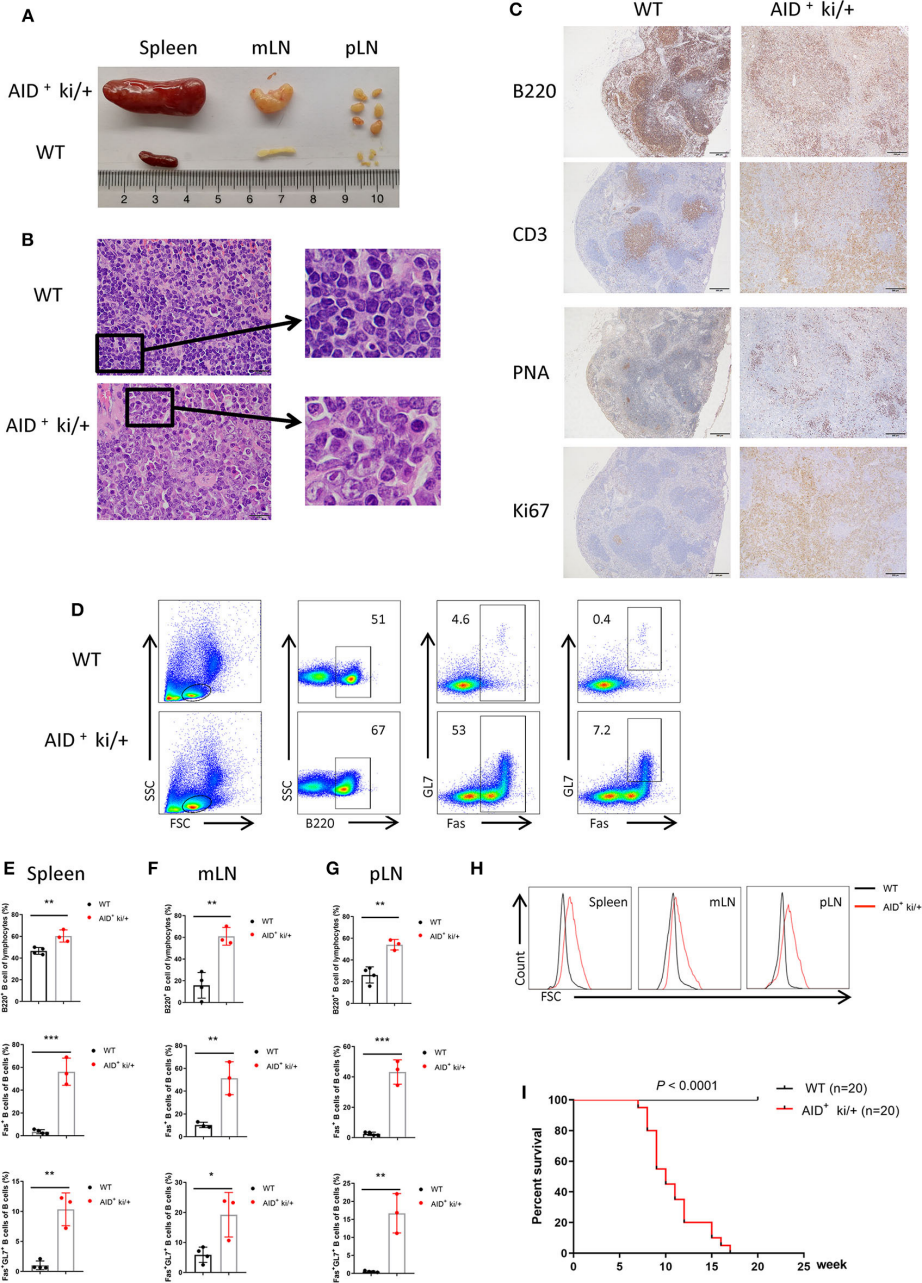
GC B cell specific c-MYC and BCL2 expression produces DLBCL phenotype. **(A)** Representative spleen, mLN and pLN from 10-week-old AID^+^ ki/+ mice and WT littermate controls. **(B)** Representative HE staining of spleen from 10-week-old AID^+^ ki/+ mice and WT controls. **(C)** Representative immunohistochemical staining (B220, CD3, PNA and Ki67) of spleen from 10-week-old AID^+^ ki/+ mice and WT controls. **(D)** Representative flow cytometry analysis of GC B cell markers (B220, GL7 and FAS) of spleen cells from AID^+^ ki/+ mice and WT controls. **(E–G)**. Mean percentage of the proportion of B cells (B220^+^), Fas^+^ B cells and GC B cells (B220^+^Fas^+^GL7^+^) of spleens **(E)**, mLN **(F)**, and pLN **(G)** from AID^+^ ki/+ mice and WT controls (*n* = 4 for WT mice and *n* = 3 for AID^+^ ki/+ mice). **(H)** Representative flow cytometry analyze the FSC of spleen, mLN and pLN B cells (B220^+^ gated in D) from AID^+^ ki/+ mice and WT controls. **(I)** Kaplan–Meier survival curve of AID^+^ ki/+ mice and WT controls (*n* = 20). Significant differences in survival were evaluated by log-rank (Mantel–Cox) test.

The *c-MYC* and *BCL2* knock-in floxed mice were generated as shown in Supplementary Figure 1A. The genotypes were identified by PCR (Supplementary Figure 1B). To overexpress c-MYC and BCL2 during GC development, we generated a conditional knock-in mouse model in which *c-MYC* and *BCL2* were co-expressed under the control of the GC B cell specific AID-Cre. GC B specific c-MYC and BCL2 expression mice (AID^+^ ki/+) and control WT mice (AID^−^ ki/+ or AID^+^ +/+) were sacrificed on 8–10 week. We observed enlarged spleen, mLN and pLN in AID^+^ ki/+ mice but not in their WT littermate controls (Figure 1A). Histopathological and immunohistochemical examination revealed that AID^+^ ki/+ mice have disruption of splenic architecture. The splenic cells from AID^+^ ki/+ mice were larger, with greater pleomorphic morphology, and also had higher Ki67 positivity, which indicated increased number of proliferating cells (Figures 1B,C). And there were lots of PNA^+^ cells in the spleen of AID^+^ ki/+ mice, while there were only a few in the WT mice (Figure 1C). The increased levels of c-MYC and BCL2 protein in the isolated splenic B cells from AID^+^ ki/+ mice than the WT mice were confirmed by western blotting (Supplementary Figure 1C). Then we analyzed the GC B cells development in the spleens by flow cytometry, and found that the proportion of B cells (B220^+^), Fas^+^ B cells (B220^+^Fas^+^) and GC B cells (B220^+^Fas^+^GL7^+^) increased significantly in the AID^+^ ki/+ mice (Figures 1D,E). Similarly, the proportion of B cells, Fas^+^ B cells and GC B cells also increased significantly in the pLN and mLN of AID^+^ ki/+ mice (Figures 1F,G). We also found larger cell size in the spleen, mLN and pLN from AID^+^ ki/+ mice by analyzing the FSC of B220^+^ B cells (Figure 1H). FITC-PNA was used to confirm the GC B cells (B220^+^Fas^+^PNA^+^) phenotypes in spleen, blood and liver from AID^+^ ki/+ mice and WT mice (Supplementary Figures 2A–C).

Additionally, a separate cohort of AID^+^ ki/+ and WT mice was followed longitudinally to assess the impact of c-MYC and BCL2 Co-expression on survival. Our results showed that the AID^+^ ki/+ mice have lower survival rate than WT controls (*n* = 20) (*P* < 0.0001). Initial death within AID^+^ ki/+ mice was reported at 7 week age, and the survival study was continue till 17 week age (Figure 1I). Overall, our data indicated that c-MYC and BCL2 Co-expression in GC B cells could induce B cell lymphoma.

### B Cell Expansion in Lung, Liver, and Kidney in c-MYC and BCL2 Co-expression Mice

**Figure 2:**
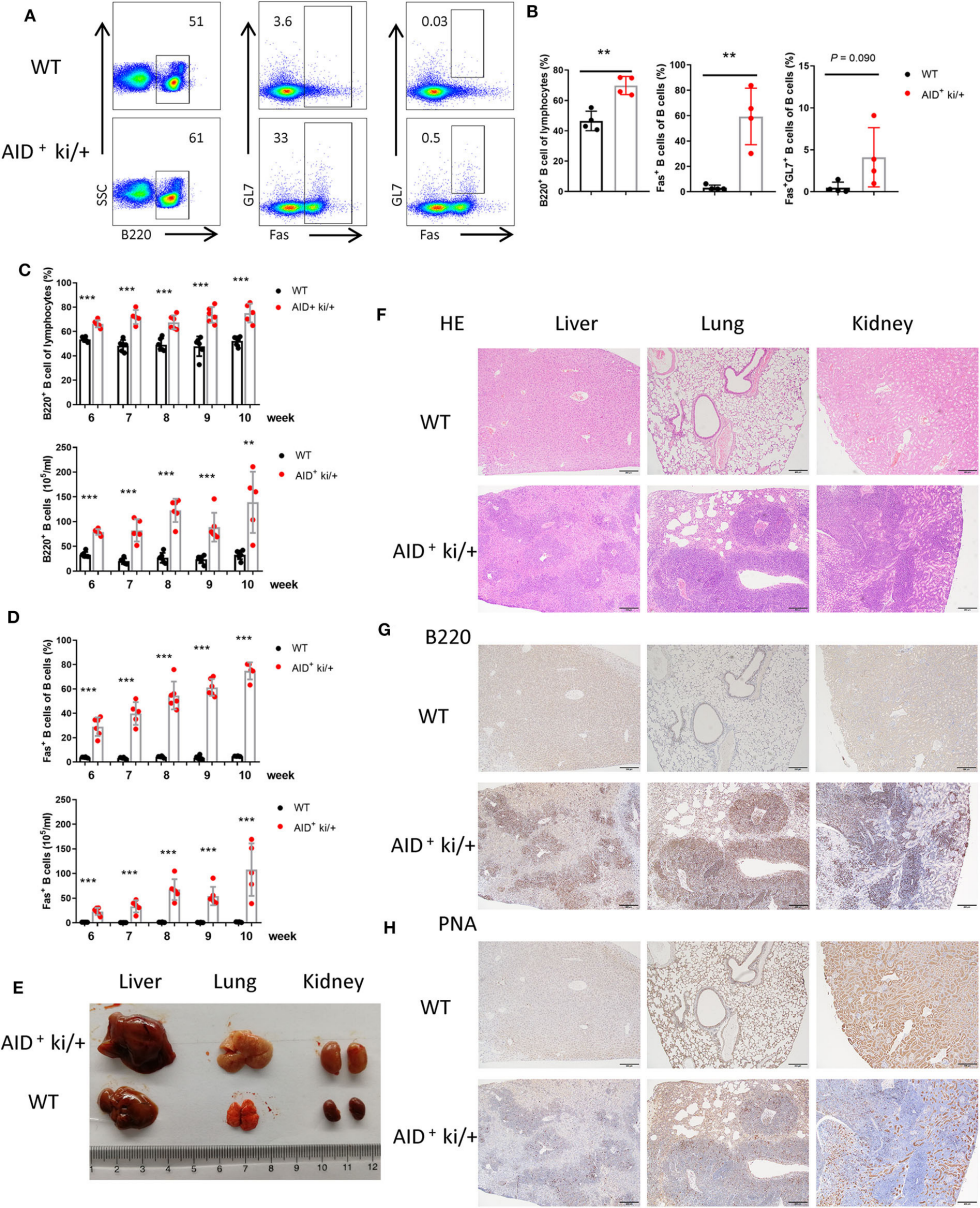
B cell expansion in the lung, liver, and kidney from blood in GC B cell specific c-MYC and BCL2 expression mice. **(A)** Representative flow cytometry analysis of GC B cell markers (B220, GL7 and FAS) of peripheral blood cells from AID^+^ ki/+ mice and WT controls. **(B)** Mean percentage of the proportion of B cells (B220^+^), Fas^+^ B cells and GC B cells (B220^+^Fas^+^GL7^+^) of peripheral blood from AID^+^ ki/+ mice and WT controls (*n* = 4). **(C,D)**. Frequency and cell concentration of B cells (B220^+^) **(C)** and Fas^+^ B cells **(D)** in the peripheral blood of 6 to 10-week-old AID^+^ ki/+ mice versus WT littermates analyzed by flow cytometry (*n* = 5–6). **(E)** Representative liver, lung and kidneny from AID^+^ ki/+ mice and WT littermate controls. **(F)** Representative HE staining of liver, lung and kidneny from AID^+^ ki/+ mice and WT controls. **(G,H)** Representative immunohistochemical staining of B220 **(G)** and PNA **(H)** of liver, lung and kidneny from AID^+^ ki/+ mice and WT controls.

Next we explored whether these tumor cells could metastasize to the other organs. We analyzed the B cells in the peripheral blood of AID^+^ ki/+ mice and WT littermate controls, and observed increased B220^+^ B cells and B220^+^Fas^+^ B cells in the AID^+^ ki/+ mice. But the proportion of GC B cells did not increase significantly (Figures 2A,B). Furthermore, a gradual increase of B220^+^ B cells and B220^+^Fas^+^ B cells were observed from 6 to 10 weeks (Figures 2C,D).

Meanwhile increscent liver, lung and kidney were observed in the AID^+^ ki/+ mice (Figure 2E). Cellular infiltration was observed in the liver, lung and kidney from AID^+^ ki/+ mice, especially around the blood vessels (Figure 2F). Immunohistochemical examination revealed that most of the infiltrated cells are B220^+^ B cells, and a small number of the infiltrated cells are PNA^+^ cells (Figures 2G,H). The increased Fas^+^ B cells population in AID+ ki/+ mice group was further confirmed by flow cytometry analysis of lymphocytes from liver, lung and kidney (Supplementary Figures 3A–C). A large population of B220^+^ B cells, most of which are Fas^+^ B cells, are found in the thymus of AID^+^ ki/+ mice (Supplementary Figures 3D,E). These data indicate that there are B cell expansion in lung, liver, and kidney in the AID^+^ ki/+ mice and the infiltrated cells may from the lymphoid organs (spleen, pLN and mLN) through the blood.

### Transplantability of c-MYC and BCL2 Co-expression Induced Lymphoma

**Figure 3:**
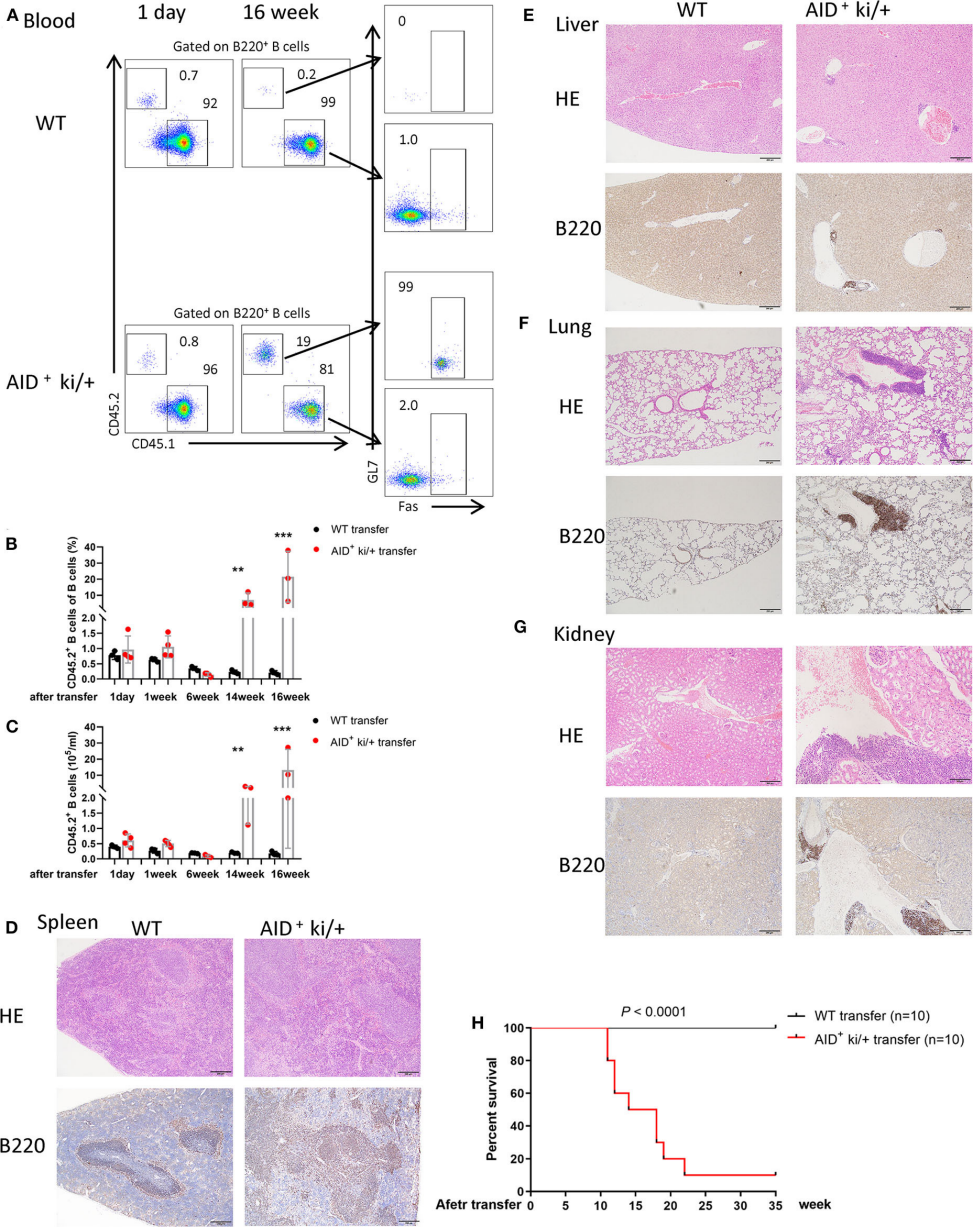
Spleen B cell of c-MYC and BCL2 expression mice transfer induced lymphoma phenotype. **(A)** Representative flow cytometry analysis of host (CD45.1) and transfer B cells (CD45.2) from peripheral blood of AID^+^ ki/+ mice and WT B cells transfer mice at 1 day or 16 weeks after transfer. **(B,C)** Mean of the proportion **(B)** and cell concentration **(C)** of transfer B cells (CD45.2) of peripheral blood from AID^+^ ki/+ mice and WT controls transfer mice at different time after transfer (*n* = 3–4). **(D–G)**. Representative, HE and immunohistochemical staining of B220 of spleen **(D)**, liver **(E)**, lung **(F)**, and kidney **(G)** from AID^+^ ki/+ mice and WT B cells transfer mice. **(H)** Kaplan–Meier survival curve of AID^+^ ki/+ mice and WT B cells transfer mice (*n* = 10). Significant differences in survival were evaluated by log-rank (Mantel–Cox) test.

We further examined the transplantability of the B cells from AID^+^ ki/+ mice. Four million splenic B cells (CD45.2/Ly 5.1) from AID^+^ ki/+ tumor-bearing mice or healthy littermate controls were injected into 8-week old BoyJ mice (CD45.1/Ly 5.2). Transplanted cells in peripheral blood were detected by flow cytometry every 2 weeks after B cell transfer. We found a similar proportion of B cells (CD45.2/Ly 5.1) from AID^+^ ki/+ mice or WT mice transfer BoyJ mice at day 1 after transfer. The proportion of B cells from AID^+^ ki/+ mice increased significantly at 14 or 16 weeks after transfer, while there was no significant change of the proportion of B cells from WT mice (Figures 3A–C). Histopathological and immunohistochemical examination showed pathological changes in spleen, and B cell expansion in the lung, liver, and kidney in AID^+^ ki/+ B cells transfer mice (Figure 3D). While there was no significant change in the WT B cells transfered mice (Figures 3E–G). Furthermore, the AID^+^ ki/+ B cells transferred mice manifested significantly shorter lifespan than the WT B cells transferred mice (Figure 3H). Significantly increased proportion of B cells in spleen, lung, liver, and kidney of AID^+^ ki/+ transferred mice was further confirmed by flow cytometry at 16 week after transfer (Supplementary Figure 4). These results suggest that the B cells from AID^+^ ki/+ mice has a strong transplantability.

### Co-expression of c-MYC and BCL2 in B Cells Could Also Induce B Cell Lymphoma

**Figure 4:**
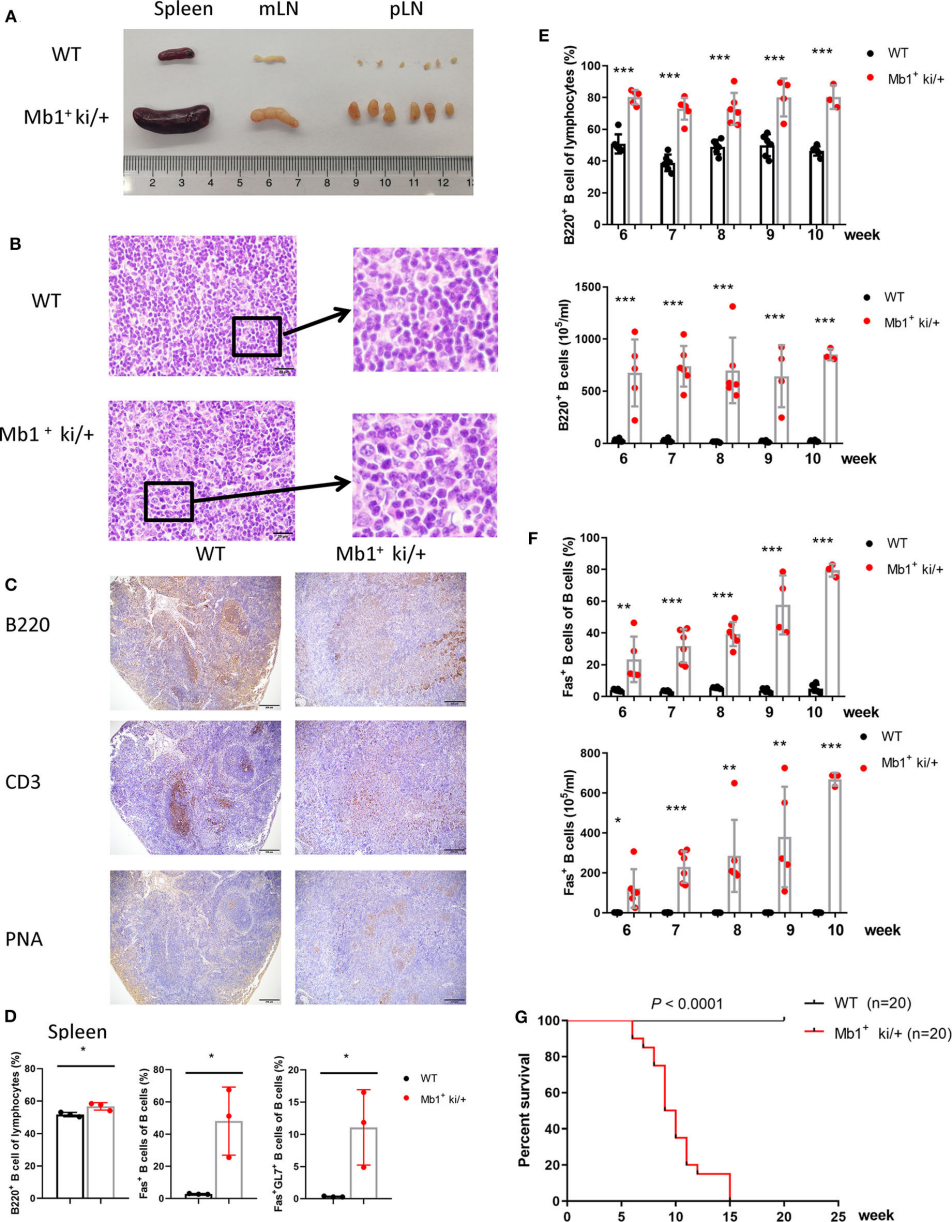
Pan-B cell specific c-MYC and BCL2 expression produces DLBCL phenotype. **(A)** Representative spleen, mLN and pLN from 10-week-old Mb1^+^ ki/+ mice and WT littermate controls. **(B)** Representative HE staining of spleen from 10-week-old Mb1^+^ ki/+ mice and WT controls. **(C)** Representative immunohistochemical staining of spleen from Mb1^+^ ki/+ mice and WT controls. **(D)** Mean percentage of the proportion of B cells (B220^+^), Fas^+^ B cells and GC B cells (B220^+^Fas^+^GL7^+^) of spleen from Mb1^+^ ki/+ mice and WT controls (*n* = 3). **(E,F)**. Frequency and cell concentration of B cells (B220^+^) **(E)** and Fas^+^ B cells **(F)** in the peripheral blood of 6 to 10-week-old Mb1^+^ ki/+ mice vs. WT littermates analyzed by flow cytometry (*n* = 3–6). **(G)** Kaplan–Meier survival curve of Mb1^+^ ki/+ mice and WT controls (*n* = 20). Significant differences in survival were evaluated by log-rank (Mantel–Cox) test.

In addition, we explored the effect of Co-expression of *c-MYC* and *BCL2* in pan-B cells by generating a conditional knockin mouse model in which c-MYC and BCL2 were co-expressed under the control of the pan-B cell specific Mb1-Cre. The expression of c-MYC and BCL2 protein in the isolated splenic B cells from Mb1^+^ ki/+ and WT mice were confirmed by western blotting (Supplementary Figure 1D). Mb1^+^ ki/+ and WT mice (Mb1^−^ ki/+ or Mb1^+^ +/+) were sacrificed on 8–10 week age. Enlarged spleens, mLNs and pLNs were observed in Mb1^+^ ki/+ mice, but not in the WT controls (Figure 4A). Histopathological and immunohistochemical examination revealed that Mb1^+^ ki/+ mice also had disruption of splenic architecture and neoplastic, and the Mb1^+^ ki/+ neoplastic cells were larger, exhibited greater pleomorphic morphology (Figures 4B,C). A large amount of PNA^+^ cells were found in the spleen of Mb1^+^ ki/+ mice, compared to the WT mice (Figure 4C). The proportion of B cells (B220^+^), Fas^+^ B cells (B220^+^Fas^+^) and GC B cells (B220^+^Fas^+^GL7^+^) also increased significantly in the Mb1^+^ ki/+ mice (Figure 4D). And the increase of B220^+^ B cells and B220^+^Fas^+^ B cells in peripheral blood showed a gradual increase from 6 to 10 weeks (Figures 4E,F). Mb1^+^ ki/+ mice manifested significantly shorter lifespan than WT control (*n* = 20) (*P* < 0.0001) (Figure 4G).

**Figure 5:**
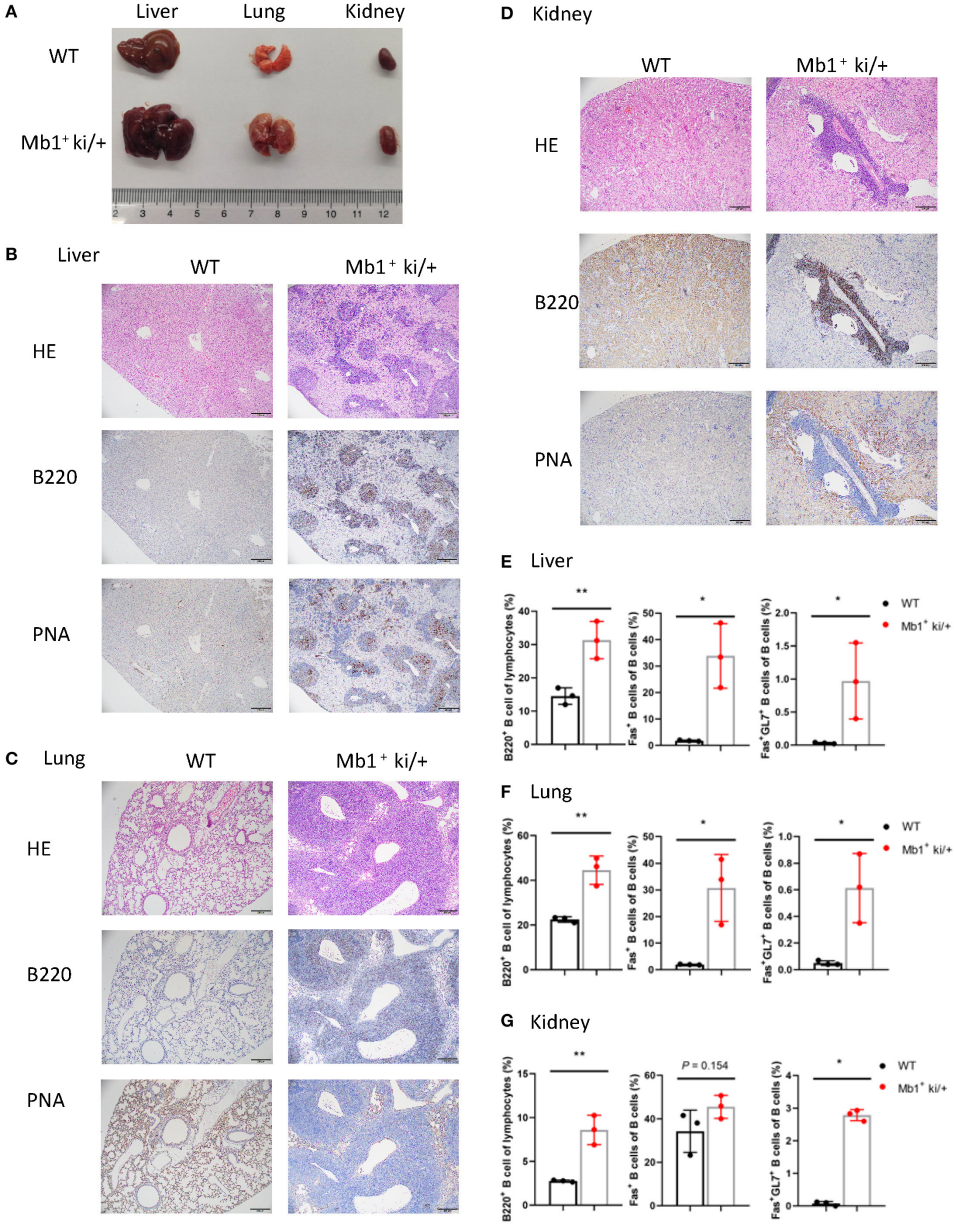
Pan-B cell specific c-MYC and BCL2 expression produces DLBCL phenotype. **(A)** Representative liver, lung and kidney from 10-week-old Mb1^+^ ki/+ mice and WT littermate controls. **(B–D)**. Representative HE and immunohistochemical staining of liver **(B)**, lung **(C)** and kidneny **(D)** from Mb1^+^ ki/+ mice and WT controls. **(E–G)** Mean percentage of the proportion of B cells (B220^+^), Fas^+^ B cells and GC B cells (B220^+^Fas^+^GL7^+^) of liver **(E)**, lung **(F)** and kidney **(G)** from Mb1^+^ ki/+ mice and WT controls (*n* = 3).

Increscent liver, lung and kidney were observed in the Mb1^+^ ki/+ mice (Figure 5A). Cellular infiltration was also found in the liver, lung and kidney from Mb1^+^ ki/+ mice. Immunohistochemical examination revealed that most of the infiltrated cells were B220^+^ B cells, and a small number of the infiltrated cells were PNA^+^ cells (Figures 5B–D). The lymphocytes in liver, lung and kidney were also analyzed by flow cytometry and higher levels of B cells, Fas^+^ B cells and GC B cells in the Mb1^+^ ki/+ mice than the WT controls were found (Figures 5E–G). In summary, these results illustrate that c-MYC and BCL2 Co-expression in pan-B cells could also induce B cell lymphoma.

## Discussion

The results described here establish a clinically relevant mouse model of double-expressor lymphoma by targeting constitutive expression of c-MYC and BCL2. We observed that Co-expression of c-MYC and BCL2 in germinal center B cells, or pan-B cells could both induce B cell lymphoma.

Lymphomas, which most frequently originate from B cells, are highly heterogeneous diseases, varying by both the type of malignant cell and the tumor location. There are two main groups of B cell lymphomas, classified as B cell Hodgkin lymphomas and non-Hodgkin lymphomas (NHL). NHL account for about 80% of all lymphomas (11), and half of the NHL are diffuse large B-cell lymphomas (DLBCL) (11).

Double-expressor lymphoma is DLBCL with Co-expression of c-MYC and BCL2 proteins by immunohistochemistry, and accounts for 21 to 34% of newly diagnosed DLBCL patients (5). Both *c-MYC* and *BCL2* are critical driver genes for NHL including DLBCL (12). The *c-MYC* protooncogene locates at chromosome 8q24 and encodes an important transcription factor which is responsible for many cellular functions including proliferation, growth, apoptosis, and differentiation (13). *BCL2* locates at chromosome 18q21 and encodes an integral outer mitochon-drial membrane protein that can inhibit cell apoptosis (14). Horn et al. found that high level of *c-MYC* expression (and/or *c-MYC* rearrangement status) in concert with BCL2 high and BCL6 low status emerged as critical prognostic variables with comparable relative risks for both event-free survival and overall survival in DLBCL (15). Patients with DEL have poor outcomes after R-CHOP induction therapy (15–18). However, the reason remains unclear.

Murine models are very useful tools to study lymphomagenesis and disease progression, as well as potential treatment in a pre-clinical setting (19). Several mouse models of B lymphoma have been successfully constructed. It was reported that combining constitutive c-MYC expression and PI3K activity in germinal center B cells of the mouse led to Burkitt lymphoma-like tumors (20). Another research found that it was not sufficient to drive neoplastic growth in BCL2 forced expression transgenic mice, but several SRBC challenges resulted in re-entry of memory B cells into the GC reaction (21). *Kmt2d* conditional deletion by Cγ1-Cre is also insufficient for transformation under continuous SRBC challenge, but lymphomagenesis was promoted when cooperated with BCL2 in VavP-*BCL2* transgenic mice (22). In additional, *Kmt2d* knockdown in VavP-*BCL2* hematopoietic progenitor cells could reduce the latent period of lymphoma relative to VavP-*BCL2* alone when transplanted into recipient mice (23). Oncogenic *BCL6* and *EZH*2 cooperated to accelerate diffuse large B cell lymphoma (DLBCL) development when chronically challenged with SRBCs (24). Monoallelic deletion of *Crebbp* mediated by Cγ1-Cre in VavP-*BCL2* mice had a significantly increased levels of follicular lymphoma (25). But, there are still no applicable murine models for double-expressor lymphoma.

In the current study, we used genetic murine models to investigate the role of c-MYC and BCL2 proteins in B cell lymphomagenesis. We found that Co-expression of c-MYC and BCL2 in germinal center B cells could induce B cell lymphoma. The tumor-bearing mice had enlarged spleen, mesenteric lymph node, and peripheral lymph node, and B-cell expansion in the lung, liver, and kidney. And the tumor-bearing mice exhibited significantly shorter lifespan and began to die at 7 week age. Another significant outcome was that the Co-expression of c-MYC and BCL2 induced lymphoma was spontaneous and occured at an early age. In addition, the B cells from AID^+^ ki/+ tumor-bearing mice also had a strong transplantability. When we used Mb1-cre to generate a conditional knockin mouse model in which c-MYC and BCL2 were co-expressed in pan-B cells, the tumorigenicity was also confirmed. We observed that the Mb1^+^ ki/+ mice had more B cells (B220^+^) and Fas^+^ B cells than the AID^+^ ki/+ mice in the peripheral blood at the same age (as shown in Figures 2C,D, 4E,F). Furthermore, the Mb1^+^ ki/+ mice developed lymphoma and died a little earlier (began to die at 6 week age, and all died at 15 week age) compared to the AID^+^ ki/+ mice (began to die at 7 week age, and all died at 17 week age).

Taken together, we show that targeting c-MYC and BCL2 expression into mouse GC B cells or pan-B cells generates a clinically relevant mouse model of double-expressor lymphoma. The tumor-bearing mice have enlarged spleen, mLN and pLN, and B cell expansion in the lung, liver, and kidney, and also significantly shorter lifespan. This model will provide a good basis to study the pathogenesis of double-expressor lymphoma and will open new and promising approaches to designing better therapies.

## Data Availability Statement

All datasets generated for this study are included in the article/Supplementary Material.

## Ethics Statement

The animal study was reviewed and approved by the institutional animal care and use committee of Nanjing Medical University.

## Author Contributions

ZC and XW conceptualized the project and designed the experiments. ZC, MC, FW, YW, WB, and SZ performed the experiments. ZC, LZ, and XW analyzed the data and wrote the manuscript. All authors contributed to the article and approved the submitted version.

## Conflict of Interest

The authors declare that the research was conducted in the absence of any commercial or financial relationships that could be construed as a potential conflict of interest.

## Supplementary Material

Supplementary Figure 1 Generation and identification of *c-MYC* and *BCL2* conditional Co-expression mice. **(A)** Schematic diagram of the generation of *c-MYC* and *BCL2* conditional Co-expression mice. **(B)** Identification of *c-MYC* and *BCL2* knockin mice and WT mice by PCR. **(C,D)** The expression of c-MYC and BCL2 from AID^+^ ki/+ mice and WT controls **(C)**, or Mb1^+^ ki/+ mice and WT controls **(D)**.

Supplementary Figure 2 GC B cell identification by B220, GL7 and PNA. Representative flow cytometry analysis of GC B cells by B220, GL7 and PNA of spleen **(A)**, peripheral blood **(B)** and liver **(C)** from AID^+^ ki/+ mice and WT controls.

Supplementary Figure 3 Flow cytometry analysis of B-cell expansion in liver, lung and kidney. **(A–C)** Mean percentage of the proportion of B cells (B220^+^), Fas^+^ B cells and GC B cells (B220^+^Fas^+^GL7^+^) of liver **(A)**, lung **(B)** and kidney **(C)** from AID^+^ ki/+ mice and WT controls (*n* = 4). **(D,E)** Representative, flow cytometry analysis of the proportion of B cells (B220^+^), Fas^+^ B cells and GC B cells (B220^+^Fas^+^GL7^+^) of thymus from AID^+^ ki/+ mice and WT controls (*n* = 4).

Supplementary Figure 4 Flow cytometry analysis of transfered B cells in BoyJ mice. **(A–D)** Representative, flow cytometry analysis of host (CD45.1) and transfer B cells (CD45.2) from spleen, liver, lung and kidney of AID^+^ ki/+ mice and WT B cells transfer mice at 16 week after transfer. **(E–H)** Mean of the proportion of transfer B cells (CD45.2) of spleen, liver, lung and kidney of AID^+^ ki/+ mice and WT B cells transfer mice at 16 week after transfer (*n* = 4).

## References

[1] Siegel RL Miller KD Jemal A. Cancer statistics, 2019. CA Cancer J Clin. (2019) 69:7–34. 10.3322/caac.21551 30620402

[2] Gisselbrecht C Glass B Mounier N Singh Gill D Linch DC Trneny M. Salvage regimens with autologous transplantation for relapsed large B-cell lymphoma in the rituximab era. J Clin Oncol. (2010) 28:4184–90. 10.1200/JCO.2010.28.1618 20660832 PMC3664033

[3] Vaux DL Cory S Adams JM. Bcl-2 gene promotes haemopoietic cell survival and cooperates with c-myc to immortalize pre-B cells. Nature. (1988) 335:440–2. 10.1038/335440a0 3262202

[4] Dang CV. MYC on the path to cancer. Cell. (2012) 149:22–35. 10.1016/j.cell.2012.03.003 22464321 PMC3345192

[5] Swerdlow SH Campo E Pileri SA Harris NL Stein H Siebert R. The 2016 revision of the world health organization classification of lymphoid neoplasms. Blood. (2016) 127:2375–90. 10.1182/blood-2016-01-643569 26980727 PMC4874220

[6] Schmitz R Wright GW Huang DW Johnson CA Phelan JD Wang JQ. Genetics and pathogenesis of diffuse large B-cell lymphoma. N Engl J Med. (2018) 378:1396–407. 10.1056/NEJMoa1801445 29641966 PMC6010183

[7] Godfrey JK Nabhan C Karrison T Kline JP Cohen KS Bishop MR. Phase 1 study of lenalidomide plus dose-adjusted EPOCH-R in patients with aggressive B-cell lymphomas with deregulated MYC and BCL2. Cancer. (2019) 125:1830–6. 10.1002/cncr.31877 30707764

[8] Herrera AF Mei M Low L Kim HT Griffin GK Song JY. Relapsed or refractory double-expressor and double-hit lymphomas have inferior progression-free survival after autologous stem-cell transplantation. J Clin Oncol. (2017) 35:24–31. 10.1200/JCO.2016.68.2740 28034071 PMC5455688

[9] Chen C Zhai SL Zhang L Chen JJ Long XH Qin J. Uhrf1 regulates germinal center B cell expansion and affinity maturation to control viral infection. J Exp Med. (2018) 215:1437–48. 10.1084/jem.20171815 29618490 PMC5940267

[10] Huang Y Guo L Qiu J Chen X Hu-Li J Siebenlist U. IL-25-responsive, lineage-negative KLRG1(hi) cells are multipotential 'inflammatory' type 2 innate lymphoid cells. Nat Immunol. (2015) 16:161–9. 10.1038/ni.3078 25531830 PMC4297567

[11] Donnou S Galand C Touitou V Sautes-Fridman C Fabry Z Fisson S. Murine models of B-cell lymphomas: promising tools for designing cancer therapies. Adv Hematol. (2012) 2012:701704. 10.1155/2012/701704 22400032 PMC3287022

[12] Ennishi D Mottok A Ben-Neriah S Shulha HP Farinha P Chan FC. Genetic profiling of MYC and BCL2 in diffuse large B-cell lymphoma determines cell-of-origin-specific clinical impact. Blood. (2017) 129:2760–70. 10.1182/blood-2016-11-747022 28351934

[13] Nie ZQ Hu GQ Wei G Cui KR Yamane A Resch W. c-Myc is a universal amplifier of expressed genes in lymphocytes and embryonic stem cells. Cell. (2012) 151:68–79. 10.1016/j.cell.2012.08.033 23021216 PMC3471363

[14] Huang W Medeiros LJ Lin P Wang W Tang G Khoury J. MYC/BCL2/BCL6 triple hit lymphoma: a study of 40 patients with a comparison to MYC/BCL2 and MYC/BCL6 double hit lymphomas. Mod Pathol. (2018) 31:1470–8. 10.1038/s41379-018-0067-x 29785017

[15] Horn H Ziepert M Becher C Barth TF Bernd HW Feller AC. MYC status in concert with BCL2 and BCL6 expression predicts outcome in diffuse large B-cell lymphoma. Blood. (2013) 121:2253–63. 10.1182/blood-2012-06-435842 23335369

[16] Johnson NA Slack GW Savage KJ Connors JM Ben-Neriah S Rogic S. Concurrent expression of MYC and BCL2 in diffuse large B-cell lymphoma treated with rituximab plus cyclophosphamide, doxorubicin, vincristine, and prednisone. J Clin Oncol. (2012) 30:3452–9. 10.1200/JCO.2011.41.0985 22851565 PMC3454768

[17] Hu S Xu-Monette ZY Tzankov A Green T Wu L Balasubramanyam A. MYC/BCL2 protein Co-expression contributes to the inferior survival of activated B-cell subtype of diffuse large B-cell lymphoma and demonstrates high-risk gene expression signatures: a report from the international DLBCL rituximab-CHOP consortium program. Blood. (2013) 121:4021–31. 10.1182/blood-2012-10-460063 23449635 PMC3709650

[18] Perry AM Alvarado-Bernal Y Laurini JA Smith LM Slack GW Tan KL. MYC and BCL2 protein expression predicts survival in patients with diffuse large B-cell lymphoma treated with rituximab. Br J Haematol. (2014) 165:382–91. 10.1111/bjh.12763 24506200

[19] Ramezani-Rad P Rickert RC. Murine models of germinal center derived-lymphomas. Curr Opin Immunol. (2017) 45:31–6. 10.1016/j.coi.2016.12.002 28160624 PMC5449224

[20] Sander S Calado DP Srinivasan L Kochert K Zhang BC Rosolowski M. Synergy between PI3K signaling and MYC in burkitt lymphomagenesis. Cancer Cell. (2012) 22:167–79. 10.1016/j.ccr.2012.06.012 22897848 PMC3432451

[21] Sungalee S Mamessier E Morgado E Gregoire E Brohawn PZ Morehouse CA. Germinal center reentries of BCL2-overexpressing B cells drive follicular lymphoma progression. J Clin Invest. (2014) 124:5337–51. 10.1172/JCI72415 25384217 PMC4348942

[22] Zhang J Dominguez-Sola D Hussein S Lee JE Holmes AB Bansal M. Disruption of KMT2D perturbs germinal center B cell development and promotes lymphomagenesis. Nat Med. (2015) 21:1190–8. 10.1038/nm.3940 26366712 PMC5145002

[23] Ortega-Molina A Boss IW Canela A Pan H Jiang Y Zhao C. The histone lysine methyltransferase KMT2D sustains a gene expression program that represses B cell lymphoma development. Nat Med. (2015) 21:1199–208. 10.1038/nm.3943 26366710 PMC4676270

[24] Beguelin W Teater M Gearhart MD Calvo Fernandez MT Goldstein RL Cardenas MG. EZH2 and BCL6 cooperate to assemble CBX8-BCOR complex to repress bivalent promoters, mediate germinal center formation and lymphomagenesis. Cancer Cell. (2016) 30:197–213. 10.1016/j.ccell.2016.07.006 27505670 PMC5000552

[25] Zhang J Vlasevska S Wells VA Nataraj S Holmes AB Duval R. The CREBBP acetyltransferase is a haploinsufficient tumor suppressor in B-cell lymphoma. Cancer Discov. (2017) 7:322–37. 10.1158/2159-8290.CD-16-1417 28069569 PMC5386396

