# Supplementary figures and images for: Generation of a Murine Model for c-MYC and BCL2 Co-expression B Cell Lymphomas
Source: Front Oncol. 2020 Jun 30;10:1007. doi: 10.3389/fonc.2020.01007 (PMC7338593; doi:10.3389/fonc.2020.01007)

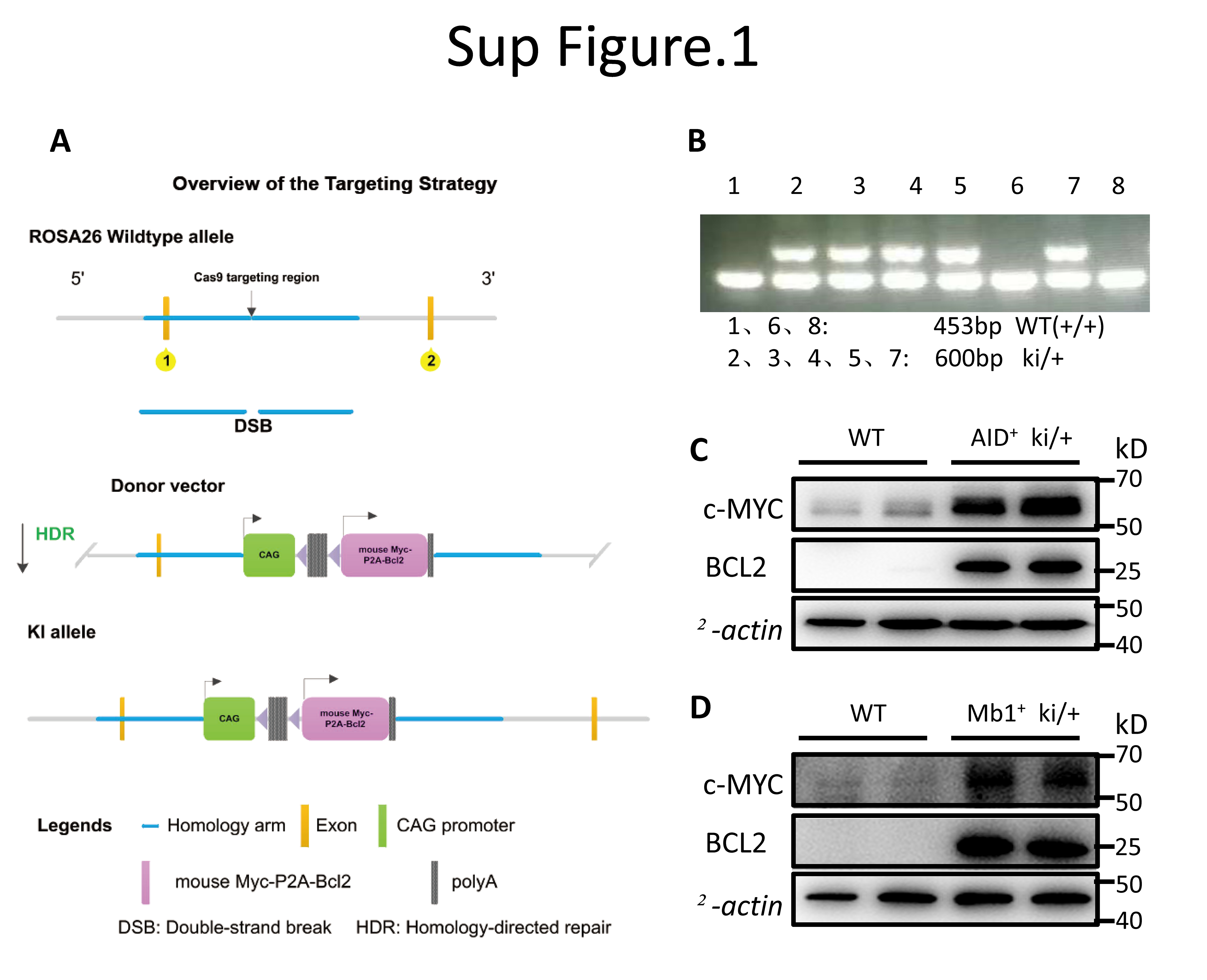

Supplement: Supplementary Figure 1 — Generation and identification of c-MYC and BCL2 conditional Co-expression mice. (A) Schematic diagram of the generation of c-MYC and BCL2 conditional Co-expression mice. (B) Identification of c-MYC and BCL2 knockin mice and WT mice by PCR. (C,D) The expression of c-MYC and BCL2 from AID+ ki/+ mice and WT controls (C), or Mb1+ ki/+ mice and WT controls (D). [file Image_1.TIFF]
